# In vitro micropropagation protocols for two endangered *Dianthus* species - via in vitro culture for conservation and recultivation purposes

**DOI:** 10.1186/s13007-025-01335-2

**Published:** 2025-02-05

**Authors:** Dóra Farkas, Judit Csabai, Angéla Kolesnyk, Pál Szarvas, Judit Dobránszki

**Affiliations:** 1 Centre for Agricultural Genomics and Biotechnology, Faculty of Agricultural and Food Science and Environmental Management, University of Debrecen, P.O. Box 12, Nyíregyháza, H-4400 Hungary; 2 Institute of Engineering and Agricultural Sciences, University of Nyíregyháza, Sóstói str. 31/b, Nyíregyháza, H-4400 Hungary; 3 Department of Genetics, Plant Biology and Microbiology, Uzhhorod National University, 3 Narodna Square, Uzhhorod, 88000 Ukraine

**Keywords:** Cytokinins, Disorders, Genotype dependence, Micropropagation, Optimal growth index

## Abstract

**Background:**

*D. giganteiformis* subsp. *pontederae* and *D. superbus* subsp. *superbus* are protected or critically endangered species in several European regions; therefore, developing an efficient in vitro micropropagation protocol is essential for germplasm conservation and recultivation purposes.

**Results:**

After germination, one-nodal segments of both species were transferred onto several MS media supplemented with 3% sucrose and different types of cytokinins (at a concentration of 4.5 µM) alongside 0.54 µM 1-naphthaleneacetic acid (NAA) for the multiplication phase for 3 weeks. The shoot clusters were subsequently transferred onto elongation medium (plant growth regulator-free MS medium) for 3 weeks. Individual shoots separated from the shoot clusters were cultured on MS medium supplemented with 0.54 µM NAA and 2% sucrose for 3 weeks for rooting. Taking into account the effects and after-effects of cytokinins, we found that the most suitable cytokinin for *D. giganteiformis* subsp. *pontederae* was N-(2-isopentenyl)-adenine (2-iP), while for *D. superbus* subsp. *superbus* it was meta-topolin (mT).

**Conclusions:**

In vitro micropropagation methods were developed for two endangered *Dianthus* species (*D. giganteiformis* subsp. *pontederae* and *D. superbus* subsp. *superbus*) by determining the optimal type of cytokinin to be used during the multiplication phase. The protocols are designed to produce large quantities of propagation material for recultivation, educational, and research purposes within three months.

## Background

Biodiversity conservation remains one of the most pressing challenges of the 21st century, demanding innovative approaches to mitigate species decline and habitat loss [1]. Among the diverse ecosystems at risk, plant species such as *Dianthus giganteiformis* Borb. subsp. *pontederae* (Kern.) Soó and *Dianthus superbus* L. subsp. *superbus*, members of the *Caryophyllaceae* family, are particularly important because of their ecological, medicinal, and conservation value [2–3]. These species exemplify the intricate interdependence between flora and fauna, serving as critical resources for pollinators such as butterflies and moths [4–6]. However, both species are increasingly threatened across Europe due to habitat degradation and anthropogenic pressures, prompting urgent conservation action [3, 7–10].

*D. giganteiformis* subsp. *pontederae*, a protected species in Hungary, is valued for its ecological role and vulnerability to extinction [11–13]. It plays a vital role as a nectar source for butterflies, such as the clouded Apollo, whose survival depends on such interdependent relationships [14–17]. On the other hand, *D. superbus* and its infraspecies, recognized for their medicinal and ecological contributions, face similar threats. Widely distributed yet critically endangered in several European regions, it is a key species in traditional medicine and is known for its diuretic, anti-inflammatory, and anticancer properties [18–23]. Its pollination by nocturnal hawkmoths emphasizes the need to protect the plant and its dependent ecological interactions [6, 24].

The use of in vitro technology for nature conservation has sparked debate [25]. However, it can be an effective tool for protecting plants, especially when traditional methods fall short [26–35]. For example, the use of seeds as a starting material often results in higher germination rates than conventional methods do [27, 36]. In cases where however, the population of a species declines to critical levels, resulting in low genetic variability, in vitro micropropagation can help conserve and restore genetic diversity, increasing the chances of survival of the species in the long term [33, 37–42]. Plants and their pollinators are interdependent. The loss of either can disrupt ecosystems. To protect pollinating insects, it is crucial to have enough preferred nectar-producing plants, and in vitro technology can help with their propagation. This underscores the need for integrating flora and fauna in conservation efforts [43–51].

Through integrated efforts focused on in vitro propagation, habitat restoration, and ecological interdependence, it is possible to address the critical threats to *D. giganteiformis* subsp. *pontederae* and *D. superbus* subsp. *superbus* while contributing to broader biodiversity conservation goals [48, 52]. Therefore, the aim of this study was to find efficient in vitro micropropagation methods for two endangered *Dianthus* species and their possible use in future applications of *ex situ* conservation, recultivation, education or research.

## Results

### Multiplication phase

After the multiplication phase, the ZEA and 2-iP treatments resulted in the significantly (*p* < 0.05) highest mean shoot length per explant (SL_M_) among all the treatments, with values of 19.03 ± 0.71 mm and 18.31 ± 0.62 mm, respectively (Table 1), in *D. giganteiformis* subsp. *pontederae*. The mean number of *de novo* shoots (SN_M_), ranged from 1.49 ± 0.13 to 5.68 ± 0.45 per explant, where ZEA resulted in the highest shoot number and the lowest shoot number was achieved in the control group (Ø). In addition to the number of newly developed shoots in the multiplication phase, one of the most important measured parameters is the number of nodes per shoot (NN_M_). The explants on KIN-containing medium presented the highest number of nodes/shoot (*p* < 0.05), with 2.85 ± 0.12 nodes/shoot. All three types of data are important for calculating the degree of shoot multiplication in a protocol. From the above three data (SL_M_, NN_M_, SN_M_), a multiplication index (MI) was calculated. On the basis of this index, we ranked the media with different CK contents: ZEA and 2-iP performed the best, with MIs of 42.06 and 31.77, respectively, followed by AD, with an MI of 27.90. These results were not convincing enough to choose the right media for the multiplication phase because the application of some CKs caused undesirable flower development, hyperhydration or necrosis of shoots to varying degrees. Therefore, other parameters, such as the survival percentage (alive percentage; A_M_%), hyperhydration percentage (H_M_%) and flowering percentage (F_M_%), were also considered. On the basis of these data, an optimal growth index (OGI_M_) was calculated for the multiplication phase, which converged to 1 and reflected the quality of the explants. In the case of ZEA and 2-iP, all explants were alive, but 32.81% and 17.14% of them, respectively, were hyperhydrated. Furthermore, 15.63% and 7.14%, respectively, had flowers. On the other hand, explants on AD-containing medium were all alive and did not flower, and only 9.23% of the explants presented signs of hyperhydration; therefore, their OGI_M_ were the highest, with a value of 0.91. Multiplying the OGI_M_ and MI indexes yielded the best medium for the multiplication phase, which encompassed all the previously mentioned parameters into a single number. Accordingly, for *D. giganteiformis* subsp. *pontederae*, the best cytokinins may be AD or 2-iP for multiplication.

**Table 1:** Mean values of measured parameters with standard errors at the multiplication phase

Treatment	SL_M_		SN_M_		NN_M_		MI	A_M_%	H_M_%	F_M_%	OGI_M_	MI * OGI_M_
***Dianthus giganteiformis*** **Borb. subsp**. ***pontederae*** (**Kern**.) **Soó**												
**Ø**	13.25 ± 0.80	c	1.49 ± 0.13	f	2.31 ± 0.13	b, c	8.55	90.91	3.64	0.00	0.87	7.46
**BA**	13.44 ± 1.07	c	2.89 ± 0.30	d, e	2.15 ± 0.15	c	18.07	83.64	12.73	1.82	0.70	12.58
**BAR**	13.15 ± 0.79	c	3.23 ± 0.25	c, d,e	2.35 ± 0.12	b, c	18.07	93.33	30.00	0.00	0.63	11.47
**KIN**	16.97 ± 0.79	a, b	3.22 ± 0.24	c, d,e	2.85 ± 0.12	a	19.17	95.00	10.00	1.67	0.84	16.02
**c**	16.82 ± 0.59	a, b	3.95 ± 0.25	b, c,d	2.68 ± 0.09	a, b	24.79	100.00	20.00	0.00	0.80	19.83
**4-CCPU**	8.09 ± 0.91	d	2.13 ± 0.24	e, f	1.27 ± 0.14	d	13.57	60.00	24.29	0.00	0.36	4.85
**2-iP**	18.31 ± 0.62	a	4.39 ± 0.37	b, c	2.53 ± 0.07	a, b,c	31.77	100.00	17.14	7.14	0.77	24.45
**ZEA**	19.03 ± 0.71	a	5.68 ± 0.45	a	2.57 ± 0.09	a, b,c	42.06	100.00	32.81	15.63	0.57	23.84
**AD**	14.94 ± 0.57	b, c	4.52 ± 0.33	a, b	2.42 ± 0.07	a, b,c	27.90	100.00	9.23	0.00	0.91	25.33
***Dianthus superbus*** **L. subsp.** ***superbus***												
**Ø**	24.23 ± 1.76	d	1.32 ± 0.12	e	1.86 ± 0.16	b, c	17.20	100.00	0.00	0.00	1.00	17.20
**BA**	45.84 ± 3.56	b, c	1.91 ± 0.14	c, d	2.94 ± 0.18	a, b,c	29.78	100.00	1.96	0.00	0.98	29.20
**BAR**	70.74 ± 3.67	a	2.94 ± 0.20	a	3.71 ± 0.18	a, b,c	56.06	100.00	0.00	0.00	1.00	56.06
**KIN**	62.71 ± 4.29	a	1.51 ± 0.12	d, e	2.71 ± 0.19	a, b,c	34.94	100.00	0.00	0.00	1.00	34.94
**mT**	57.41 ± 3.28	a, b	2.44 ± 0.18	b	3.91 ± 0.21	a, b	35.83	100.00	0.00	0.00	1.00	35.83
**4-CCPU**	35.58 ± 3.30	c, d	1.89 ± 0.14	c, d	1.71 ± 0.24	c	39.33	100.00	2.00	0.00	0.98	38.54
**2-iP**	67.50 ± 3.52	a	1.57 ± 0.08	d, e	3.06 ± 0.24	a, b,c	34.63	100.00	0.00	20.00	0.80	27.71
**ZEA**	61.32 ± 4.04	a	2.18 ± 0.17	b, c	4.78 ± 1.54	a	27.97	100.00	0.00	0.00	1.00	27.97
**AD**	28.31 ± 2.38	d	1.15 ± 0.05	e	2.49 ± 0.14	b, c	13.07	100.00	0.00	0.00	1.00	13.07

The letters following the measurements indicate significantly (*P* < 0.05) different values between treatments according to ANOVA and Tukey tests (abbreviations: Ø = control group, SL_M_= shoot length (mm); SN_M_ = new shoot number/explant; NN_M_ = node number/shoot; MI = Multiplication Index; A_M_ = alive percentage (%); H_M_ = hyperhydration percentage (%); F_M_ = flowering percentage (%); OGI_M_ = Optimal Growth Index; M in lower index = multiplication phase)

In the case of *D. superbus* subsp. *superbus*, the highest shoot length (SL_M_) was achieved on BAR (70.74 ± 3.67 mm), 2-iP (67.50 ± 3.52 mm), KIN (62.71 ± 4.29 mm) and ZEA (61.32 ± 4.04 mm), but these numbers were not significantly different from one another. The highest shoot number per explant (SN_M_) was recorded on BAR (2.94 ± 0.20), and the lowest was recorded on the control medium (1.30 ± 0.12); the last one did not differ significantly from KIN (1.51 ± 0.12) or 2-iP (1.57 ± 0.08). ZEA performed the best in terms of the number of nodes per shoot (NN_M_), with a value of 4.78 ± 1.54, but this outcome was not significantly different from those of mT, BAR, BA, 2-iP and KIN. From these data, the highest MI (56.06) was calculated for BAR. With respect to the other parameters from which the OGI_M_ was calculated, BAR, KIN and mT exceeded all the expectations: all explants were alive, and none of them were hyperhydrated or flowered on these media. Considering the values of MI * OGI_M_ for each CK, the best CK for the multiplication phase may be BAR, followed by KIN, mT and 4-CCPU, for *D. superbus* subsp. *superbus* (Table 1).

### Elongation phase

The effects of different CKs applied in the multiplication phase were evaluated at the end of the elongation phase (Table 2). In terms of shoot length (SL_E_), the highest *D. giganteiformis* subsp. *pontederae* shoots were recorded after multiplication on BA (27.36 ± 1.18 mm) and BAR (24.78 ± 1.22 mm), which were not significantly different from each other. The node number per shoot (NN_E_) was considerably but not significantly greater for AD (3.93 ± 0.12) than for any other treatment group, except for mT (3.40 ± 0.12) and 2-iP (3.14 ± 0.11). Thus, the highest elongation index (EI) was achieved for the BA (7.07) treatment, followed by the BAR (6.49) and ZEA (6.29) treatments. With respect to the parameters included in the OGI_E_ number, all of the previously mentioned groups performed poorly, with more than 30% (BA) or 18% (ZEA) flowering and signs of hyperhydration (8.89% for BAR, 4% for ZEA), resulting in 0.87, 0.79 and 0.69 OGI_E_ numbers for BAR, ZEA and BA, respectively. By multiplying the two indexes, the 2-iP treatment group performed the best, with a value of 5.70, whereas the AD group achieved a value of only 4.59 because of the high flowering rate (11.67%) and hyperhydration rate (1.67%).

**Table 2:** Mean values of measured parameters with standard errors at the elongation phase

Treatment	SL_E_		NN_E_		EI	A_E_%	H_E_%	F_E_%		OGI_E_		EI * OGI_E_
***Dianthus giganteiformis*** **Borb. subsp**. ***pontederae*** (**Kern**.) **Soó**												
**Ø**	19.51 ± 0.92	d	3.53 ± 0.14	a, b,c	5.53	100.00	0.00	0.00		1.00		5.53
**BA**	27.36 ± 1.18	a	3.87 ± 0.11	a, b	7.07	100.00	0.00	30.91		0.69		4.88
**BAR**	24.78 ± 1.22	a, b	3.82 ± 0.10	a, b	6.49	100.00	8.89	4.44		0.87		5.65
**KIN**	17.86 ± 0.83	d	3.48 ± 0.12	a, b,c	5.13	100.00	0.00	0.00		1.00		5.13
**mT**	18.16 ± 0.74	d	3.40 ± 0.12	b, c	5.34	97.14	0.00	1.43		0.96		5.11
**4-CCPU**	21.00 ± 0.98	c, d	3.42 ± 0.10	a, b,c	6.14	100.00	13.33	6.66		0.81		4.97
**2-iP**	18.26 ± 0.79	d	3.14 ± 0.11	c	5.82	100.00	0.00	2.00		0.98		5.70
**ZEA**	23.66 ± 0.89	b, c	3.76 ± 0.12	a, b	6.29	100.00	4.00	18.00		0.79		4.95
**AD**	20.75 ± 0.74	c, d	3.93 ± 0.12	a	5.28	100.00	1.67	11.67		0.87		4.59
***Dianthus superbus*** **L. subsp.** ***superbus***												
**Ø**	46.88 ± 3.14	c, d	3.66 ± 0.21	a, b,c	12.81	100.00	0.00	0.00		1.00		12.81
**BA**	57.46 ± 4.02	b, c	3.83 ± 0.24	a, b,c	15.00	100.00	0.00	12.00		0.88		13.20
**BAR**	86.36 ± 3.59	a	4.57 ± 0.19	a	18.90	100.00	0.00	12.73		0.87		16.49
**KIN**	71.57 ± 4.45	a, b	3.57 ± 0.21	a, b,c	20.05	100.00	0.00	27.27		0.73		14.58
**mT**	57.86 ± 3.70	b, c	3.97 ± 0.20	a, b	14.57	100.00	1.82	3.63		0.95		13.79
**4-CCPU**	45.09 ± 3.99	c, d	2.34 ± 0.23	d	19.27	96.36	3.64	3.64		0.89		17.24
**2-iP**	80.93 ± 4.36	a	2.86 ± 0.18	c, d	28.30	100.00	0.00	18.18		0.82		23.15
**ZEA**	71.85 ± 4.80	a, b	3.72 ± 0.26	a, b,c	19.31	92.73	0.00	45.45		0.51		9.77
**AD**	35.69 ± 3.18	d, e	2.86 ± 0.71	c, d	12.48	100.00	0.00	0.00		1.00		12.48

The letters following the measurements indicate significantly (*P* < 0.05) different values between treatments according to ANOVA and Tukey tests (abbreviations: Ø = control group, SL_E_= shoot length (mm); NN_E_ = node number/shoot; EI = Elongation Index; A_E_ = alive percentage (%); H_E_ = hyperhydration percentage (%); F_E_ = flowering percentage (%); OGI_E_ = Optimal Growth Index; E in lower index = elongation phase)

The highest shoots of *D. superbus* subsp. *superbus* at the elongation phase was recorded for the BAR treatment (86.36 ± 3.59 mm), followed by the 2-iP (80.93 ± 4.36), KIN (71.57 ± 4.45) and ZEA (71.85 ± 4.80) treatments, which did not differ significantly from one another. The mean number of nodes per shoot ranged from 2.34 ± 0.23 (4-CCPU) to 4.57 ± 0.19 (BAR), the latter of which was not significantly different from most of the CKs used earlier in the multiplication phase except 4-CCPU and 2-iP. With a 28.30 EI number, the 2-iP group performed the best compared with the other CKs, but with respect to the flowering percentage (18.18%), a new ranking was established. BAR and KIN also caused flowering in 12.73% and 27.27% of the shoots, respectively, whereas only 96.36% of the explants remained alive after the 4-week-long elongation phase following the 4-CCPU treatment. In this state, the best after-effects are caused by mT, with low percentages of hyperhydration (1.82%) and flowering (3.63%) while also meeting the quantitative expectations (Table 2).

### Rooting and acclimation phase

The experiments revealed that cytokinins had an after-effect not only in the elongation phase but also in the rooting phase. The *D. giganteiformis* subsp. *pontederae* explants that originated from the 4-CCPU (6.58 ± 0.60), BA (5.04 ± 0.60) and 2-iP (4.98 ± 0.59) multiplication media presented the highest root number (RN), while there was no significant difference between the treatment groups. The root length (RL) of the plants in the 4-CCPU (28.89 ± 2.57) and BA (16.91 ± 2.30) groups was outstanding. The rooting percentage after KIN-containing multiplication medium was the lowest (37.80%), whereas that of 4-CCPU-containing multiplication medium was the highest (90.00%). The consequence of these results was that the best RI value was accomplished as an after-effect of 4-CCPU (172.99), followed by BA (68.18) and 2-iP (59.60). However, considering these three treatments, the OGI_R_ value was the best for 2-iP, as there were no undesirable after-effects following its application in the multiplication phase, whereas offshoots that developed on BA and 4-CCPU-containing multiplication media presented signs of hyperhydration and flowering. In addition to the high RI and OGI_R_ values, 2-iP had the best after-effect compared with the other treatment groups, with no side effects.

With 16.42 ± 1.53 roots, the control group performed significantly well in the case of *D. superbus* subsp. *superbus* explants, followed by the mT (10.50 ± 1.08) and BA (7.84 ± 1.22) groups. The same tendency was observed for root length: the control group presented a length of 24.70 ± 3.15 mm, followed by the mT (21.23 ± 2.95 mm) and BA (14.33 ± 2.82 mm) groups, with no significant difference between these groups. Only two treatment groups exceeded 80.00% rooting percentage: the control group, with 88.00%, and the mT group, with 81.64%; therefore, these two groups also had the highest RI and OGI_R_ values (Table 3).

**Table 3:** Mean values of measured parameters with standard errors at the rooting phase

Treatment	RN		RL		RP%	A_*R*_%	H_*R*_%	F_*R*_%	RI	OGI_*R*_	RI *OGI_*R*_
***Dianthus giganteiformis*** **Borb. subsp**. ***pontederae*** (**Kern**.) **Soó**											
**Ø**	2.80 ± 0.44	b, c	11.10 ± 2.30	b, c	58.00	100.00	2.00	0.00	18.03	0.98	17.67
**BA**	5.04 ± 0.60	a, b	16.91 ± 2.30	a, b	80.00	100.00	1.82	3.64	68.18	0.95	64.50
**BAR**	3.55 ± 0.58	b, c	15.04 ± 2.82	b, c	60.00	100.00	1.82	5.45	32.04	0.93	29.74
**KIN**	1.93 ± 0.49	c	5.76 ± 1.72	c	37.80	100.00	0.00	0.00	4.20	1.00	4.20
**mT**	2.78 ± 0.47	b, c	11.05 ± 2.22	b, c	45.00	100.00	0.00	1.66	13.82	0.98	13.59
**4-CCPU**	6.58 ± 0.60	a	28.89 ± 2.57	a	91.00	100.00	1.82	1.82	172.99	0.96	166.75
**2-iP**	4.98 ± 0.59	a, b	14.96 ± 2.31	b, c	80.00	100.00	0.00	0.00	59.60	1.00	59.60
**ZEA**	3.53 ± 0.60	b, c	12.75 ± 2.56	b, c	62.50	100.00	0.00	5.00	28.13	0.95	26.72
**AD**	2.92 ± 0.51	b, c	11.76 ± 2.34	b, c	62.00	100.00	0.00	2.00	21.29	0.98	20.86
***Dianthus superbus*** **L. subsp.** ***superbus***											
**Ø**	16.42 ± 1.53	a	24.70 ± 3.15	a	88.00	100.00	0.00	0.00	356.91	1.00	356.91
**BA**	7.84 ± 1.22	b, c	14.33 ± 2.82	b, c	60.00	100.00	0.00	7.27	67.41	0.93	62.51
**BAR**	2.75 ± 0.53	d	7.15 ± 1.30	c, d	43.64	100.00	0.00	9.09	8.58	0.91	7.80
**KIN**	3.48 ± 0.95	d	10.62 ± 2.70	b, c,d	40.00	100.00	0.00	4.00	14.78	0.96	14.19
**mT**	10.50 ± 1.08	b	21.23 ± 2.95	a, b	81.64	98.33	0.00	0.00	181.99	0.98	178.95
**4-CCPU**	4.31 ± 0.69	c, d	12.25 ± 1.99	b, c,d	52.73	85.46	0.00	18.08	27.84	0.70	19.49
**2-iP**	1.73 ± 0.64	d	3.03 ± 1.06	d	18.33	93.33	0.00	16.67	0.96	0.78	0.75
**ZEA**	3.47 ± 0.82	d	13.65 ± 3.36	b, c,d	36.36	100.00	0.00	32.73	17.22	0.67	11.59
**AD**	3.16 ± 0.59	d	6.13 ± 1.31	c, d	52.73	100.00	0.00	1.82	10.21	0.98	10.03

The letters following the measurements indicate significantly (*P* < 0.05) different values between treatments according to ANOVA and Tukey tests (abbreviations: Ø = control group, RN = root number, RL = root length, RI = Rooting Index; RP% = rooting percentage, A_R_ = alive percentage (%); H_R_ = hyperhydration percentage (%); F_R_ = flowering percentage (%); OGI_R_ = Optimal Growth Index; R in lower index = rooting phase)

After the 2-week acclimation process, the percentages of surviving and further developed plants were high. It was determined that 90.90% of the *D. superbus* subsp. *superbus* plantlets (originated from the 2-iP medium) survived and developed, whereas in the case of *D. giganteiformis* subsp. *pontederae*, the acclimation percentage was 89.09% (originated from the mT medium).

### Disorders and undesirable developmental traits

**Fig. 1:**
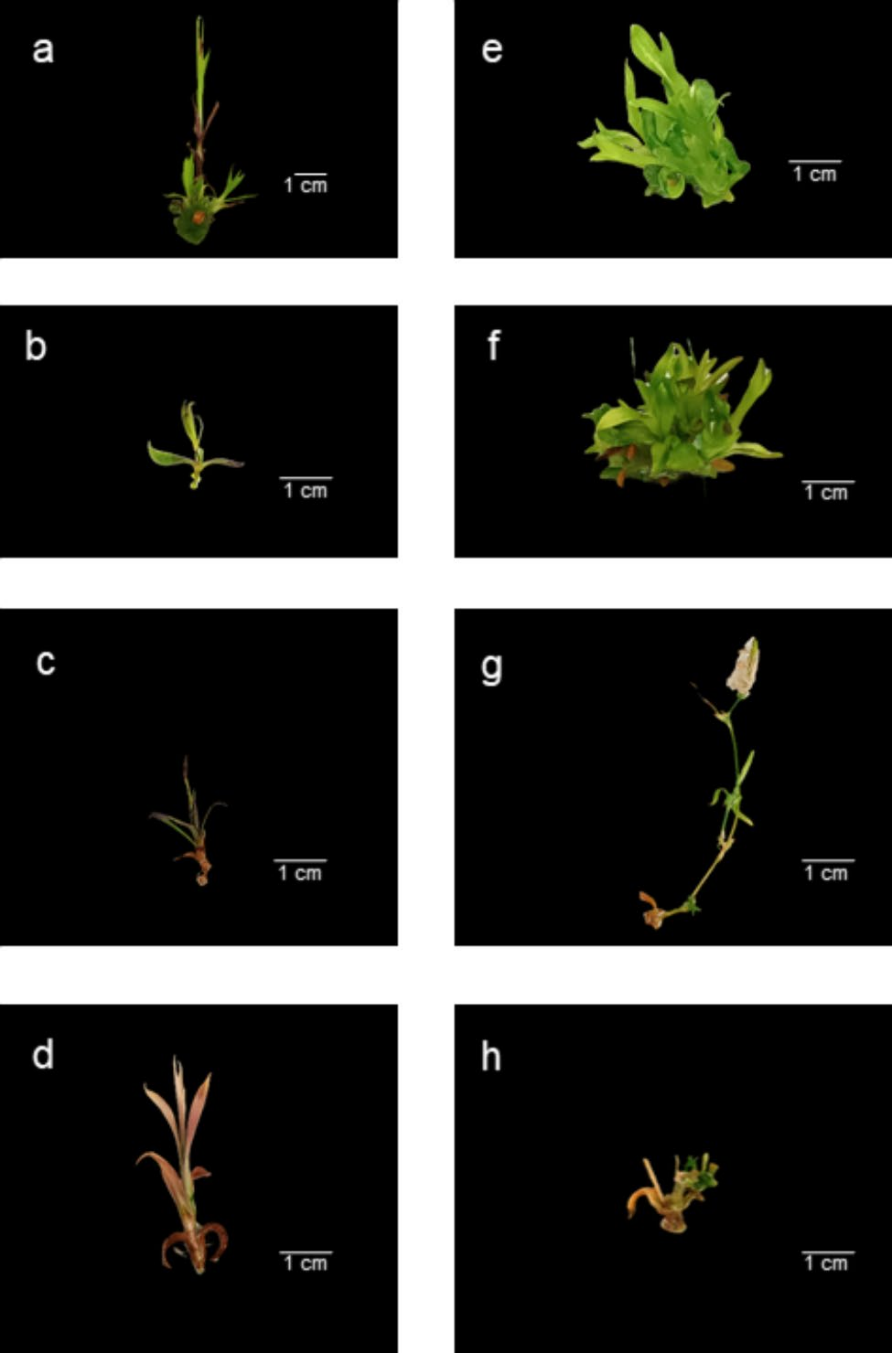
Disorders and undesirable developmental traits caused by various cytokinins applied in the multiplication medium. Direct effects in the shoot multiplication phase (**a, b, e, f**) or after-effects in the elongation (**c, h**) or in vitro rooting (**d, g**) phase were recorded. In the case of *D. giganteiformis* subsp. *pontederae* (**a-d**) at the multiplication phase, in the presence of mT, callus formation and anthocyanin production (**a**) while using ZEA, stunted growth (**b**) was observed. Furthermore, after ZEA, necrosis (**c**) occurred during the elongation phase, and after 4-CCPU, discolored explants arose during the rooting phase (**d**). For *D. superbus* subsp. *superbus* (**e-h**) hyperhydrated, bushy plants appeared to be a direct effect of BAR (**e**), and bushy and necrotic explants were also observed at the multiplication phase when BA was used in the medium (**f**). During the rooting phase, flowering also occurred as an after-effect of 2-iP (**g**), whereas stunted growth was recorded at the elongation phase when KIN was used previously (**h**). Importantly, only a few expressive cases of disorders or undesirable developmental traits are highlighted in this figure

As discussed in the previous chapters, often as a direct or delayed after-effects of CKs, some disorders or undesirable developmental traits were observed during the experiments. These properties include hyperhydration, stunted or bushy shoot growth, shoot tip necrosis and anthocyanin accumulation in the leaves. Flowering was also observed in some CK-supplemented multiplication media or as an after-effect of some CKs during the elongation or even rooting phase (Tables 1, 2 and 3; Fig. 1). Some of these traits are illustrated in Fig. 1.

## Discussion

To choose the right cytokinin for in vitro propagation, not only the direct cytokinin effect but also the subsequent after-effects must be taken into account [53–54].

During the multiplication phase, for *D. giganteiformis* subsp. *pontederae*, MS medium supplemented with 3% sucrose, 0.54 µM NAA, and 4.5 µM AD or 2-iP was the most suitable culture medium on the basis of the obtained and calculated multiplication data. Markovic et al. [32] examined another subspecies (*D. giganteiformis* Borbas subsp. *kladovanus* (Degen) Soó) of the same species (*D. giganteiformis* (Borbás) Heinr. Braun) reported that MS medium containing 0.1 mg/L BA (0.44 µM) and 0.1 mg/L NAA (0.53 µM) was the most suitable medium for shoot multiplication. Compared with the culture medium found to be the best by Markovic et al. [32], when one nodal segment of the *D. giganteiformis* subsp. *kladovanus* subspecies was used, the number of shoots was 3.6 per explant, whereas in our study, for the AD and 2-iP treatments, in both cases, the shoot number was greater (4.52 and 4.39, respectively). The node number per shoot was 2.36 for the *D. giganteiformis* subsp. *kladovanus* subspecies, whereas in the present study, 2.42 and 2.53 were the mean values of this measured parameter in AD and 2-iP, respectively. However, Markovic et al. [32] did not examine the after-effects of the CK treatments. Nevertheless, if we also consider the after-effects of CKs, it is possible to determine the most suitable medium for plant tissue culture more accurately and precisely. For example, in our present study, for shoot multiplication of *D. giganteiformis* subsp. *pontederae* AD seemed to be the most suitable CK; however, severe after-effects occurred during the elongation phase. A total of 11.67% of the explants flowered, and 1.67% of them presented signs of hyperhydration. In contrast, 2-iP caused only 2.00% of the shoots to flower, and no hyperhydration occurred at the elongation phase; moreover, no undesirable after-effects were recorded in the rooting phase (Tables 1, 2 and 3).

With respect to *D. superbus*, only a few scientific studies have been conducted on optimizing in vitro conditions for the micropropagation of this species, since these studies were published primarily in relation to callus cultures. Kim et al. [55] accomplished successful plant regeneration from leaf mesophyll protoplasts of *D. superbus;* however, the primary purpose of the protocol was to identify potential germplasm sources for somatic hybridization. Lee et al. [56] established a successful plant regeneration system from shoot tip-derived embryogenic calli at a comparatively high frequency of *D. superbus*. Holobiuc et al. [57] developed conservation and medium-term micropropagation protocols for some *Catyophyllaceae* endemic species, including *D. superbus* L. subsp. *alpestris* Kablik ex Čelak, but did not provide quantified data, except for axillary shoot formation. The same can be said of Osvalde et al.’s [34] study, in which no specific results were reported on the in vitro micropropagation of the species, mainly not for the subspecies.

Considering the calculated MI * OGI_M_ values after the multiplication phase, the most suitable CKs for the micropropagation of *D. superbus* were BAR, KIN, mT and 4-CCPU. Unfortunately, BAR and KIN had unpleasant after-effects (high hyperhydration percentages of 27.27% and 12.73%, respectively) after the elongation phase, resulting in relatively low OGI_E_ values. During this phase, there was not a large difference between the EI * OGI_E_ values of 4-CCPU (17.24) and mT (13.79), but with respect to 4-CCPU, some of the explants showed necrosis or even died (96.36% survival percentage). 4-CCPU caused excessive flowering in 18.08% of the explants examined, whereas in the case of mT, no hyperhydration or flowering was recorded after the rooting phase (Tables 1, 2 and 3; Fig. 1).

The acclimation percentage was high in both cases (90.90% for *D. giganteifomis* subsp. *pontederae* and 89.09% for *D. superbus* subsp. *superbus*). For other *Dianthus* taxa, similar results were reported. For *Dianthus caryophyllus* L. cultivars, the acclimation percentage when a pasteurized soil mixture consisting of sand, leaf-mold and vermiculite (1:1:1, v/v/v) was used can reach 90% [58], whereas for *Dianthus petraeus* Waldst. & Kit., 90–100% of the plantlets survived this process [59]. *Dianthus pinifolius* Sm. achieved an 88.9% acclimation rate on sterilized substrate made from ground/perlite in a 1:1 ratio, whereas *Dianthus trifasciculatus* subsp. *parviflorus* Stoj.& Acht. reached ~ 80% [60], *Dianthus mainensis* Shaulo & Erst reached 83% on a sand: vermiculite mixture (1:1) [61], and *Dianthus fruticosus* L. reached only 70% [62].

**Fig. 2:**
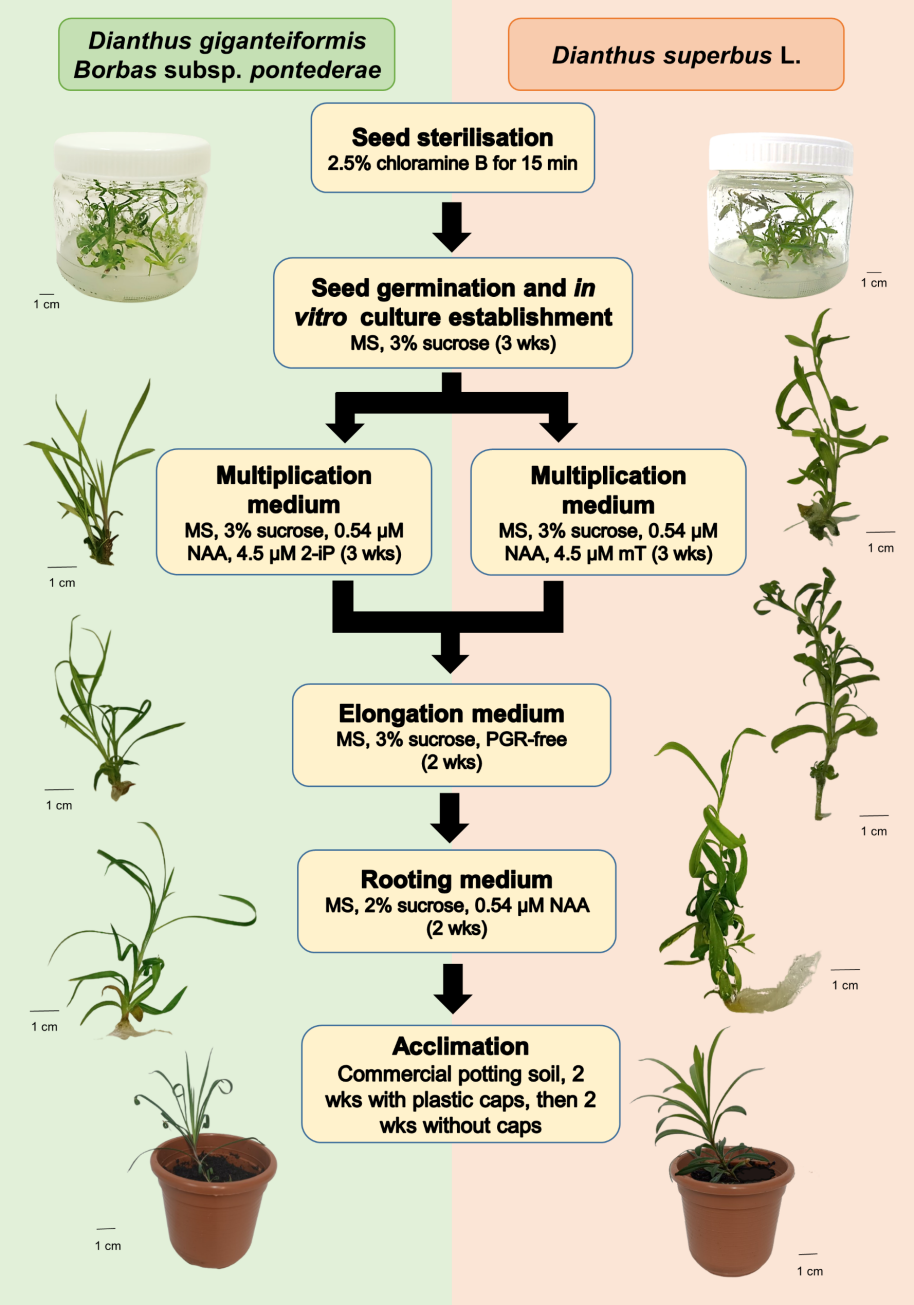
In vitro micropropagation protocols for *D. giganteiformis* subsp. *pontederae* and *D. superbus* subsp. *superbus*

In this study, we successfully developed an optimized in vitro micropropagation method (Fig. 2) for both species. For *D. giganteiformis* subsp. *pontederae*, the optimal in vitro medium for shoot multiplication was MS supplemented with 3% sucrose, 0.54 µM NAA, and 4.5 µM 2-iP, whereas MS supplemented with 3% sucrose, 0.54 µM NAA, and 4.5 µM mT was the right choice for *D. superbus* subsp. *superbus*. For the elongation phase, the optimal medium was PGR-free MS with 3% sucrose, whereas MS with 2% sucrose and 0.54 µM NAA was suitable for in vitro rooting in both cases. For the acclimation process, commercial potting soil and tap water were sufficient, provided that the plants were covered with plastic bags for the first two weeks. The detailed protocols are illustrated in Fig. 2.

## Conclusion

It is essential to emphasize holistic approaches to preserving protected plants and habitats. We recommend the application of micropropagation not only for protected plant species with weak seed germination capacity, populations below the extinction threshold, or those with significant ornamental horticultural or medicinal value but also in consideration of ecological food chains, ecosystems, habitat structures, interdependencies, and the population size of the protected plants. Even in cases where the disappearance of a protected plant species disrupts the ecosystem or food chain, it is worth increasing their numbers, potentially through vegetative propagation, i.e., application of in vitro micropropagation.

In this study, for two endangered *Dianthus* species/subspecies two effective and rapid in vitro subspecies-adjusted micropropagation protocols were developed (Fig. 2). The optimal type of cytokinins in the shoot multiplication medium was determined for both species, which allowed efficient micropropagation to be carried out most effectively with the least harmful side effects in the shoot multiplication phase or after-effects in the elongation and rooting phases. For *D. giganteiformis* subsp. *pontederae* 2-iP, while for *D. superbus* subsp. *superbus*, mT was the optimal cytokinin type when it was applied at a concentration of 4.5 µM, together with 0.54 µM NAA in the shoot multiplication medium. The protocols are suitable for rapid plant regeneration and for producing large amounts of propagation material within 3 months for recultivation purposes. The obtained results presented the average response to culture *in vitro.*

## Materials and methods

### Establishment of in vitro cultures

The present study was carried out at the Centre for Agricultural Genomics and Biotechnology, Faculty of Agricultural and Food Science and Environmental Management, University of Debrecen, Hungary, from March 2024 to the end of July 2024. The experiment began in 2022 with seeds collected from a natural population of *D. giganteiformis* subsp. *pontederae* in Hungary, specifically from Budapest, Budaörs, and Odvas-hegy (N 47°28’5.66” E 18°56’47.87”), and preserved in the collection of the Botanical Garden Leipzig (IPEN number: HU-0-KL-2012/785, 2022). The seeds of *D. superbus* subsp. *superbus* used for in vitro culture originated from the Klagenfurt Botanical Garden (*D. superbus* subsp. *superbus* * AT-1-KL-2014/3364-Austria: Carinthia, Grafenstein, Sabuatach; N 46°35’21.3” E 14°28’03.7” [± 10 m]).

The seeds underwent no prior treatments, including abrasion, stratification, or any chemical enhancement methods for germination, prior to their introduction in vitro. We used only surface sterilization of viable seeds, which was carried out according to Cseh et al. [63]. Germination was carried out on Murashige and Skoog [64] medium (MS medium) supplemented with 6.5 g/L agar (Merck-Sigma Aldrich, A1296 Plant Agar) and 3% sucrose according to Szarvas et al. [54]. 70 ml medium was poured into each culture vessel (400 ml jars) covered with plastic caps. The vessel type and the medium quantity/vessel were always the same throughout all the in vitro phases. For the germination and establishment phase, plant growth regulators (PGRs) were not added to the media. The pH was adjusted to 5.8 before autoclaving at 121 °C and 1.2 bar pressure for 15 min. All cultures (including seeds, seedlings and plants) were maintained under controlled conditions inside a culture room with a 16/8 photoperiod at a light intensity of 80–106 µmol s^− 1^ m^− 2^, provided by a 1:1 ratio of warm white and daylight fluorescent lamps at a temperature of 23 ± 2 °C. After 3 weeks of cultivation, the seedlings of both species were transferred onto fresh media several times to secure an adequate amount of plant material for the experiments.

### Multiplication and elongation phases in vitro

The composition of the multiplication medium was almost identical to that of the medium used for germination and establishment and was modified only by the addition of PGRs according to Szarvas et al. [54] before autoclaving. The MS medium was supplemented with 3% sucrose and 8 types of cytokinins (CKs, namely, 6-benzylaminopurine (BA), 6-benzylaminopurine riboside (BAR), kinetin (KIN), meta-topolin (mT), N-(2-chloro-4-pyridyl)-N’-phenylurea (4-CPPU), N-(2-isopentenyl) adenine (2-iP), zeatin (ZEA), adenine sulfate (AD), respectively) alone, at a concentration of 4.5 µM along with 0.54 µM 1-naphthaleneacetic acid (NAA). For the control group (Ø), MS medium was supplemented with 3% sucrose and 0.54 µM NAA. Five 5 explants were placed in each culture vessel.

The culture period lasted 3 weeks, after which the shoot clusters were transferred onto PGR-free MS medium (5 cluster per jar), which served as elongation medium, for 3 weeks.

### Rooting phase in vitro and acclimation

Individual shoots separated from the shoot clusters were cultured on full-strength MS medium supplemented with 0.54 µM NAA, 2% sucrose and 6.5 g l^− 1^ plant agar for 3 weeks (5 shoots per vessel).

The rooted plantlets originated from the best performing cytokinin-containing multiplication medium in each species, respectively, were transferred onto commercial garden soil (Mr. Garden, Agro CS Hungary Ltd.) after careful washing of the remaining media from the roots with normal tap water. The acclimation process lasted 4 weeks at a temperature of 23 ± 2 °C. During this period, the plantlets were irrigated with tap water regularly to ensure adequate moisture levels, and to maintain humidity, the pots were covered with plastic caps for the first 2 weeks. The climate room, where the acclimation process took place, was equipped with daylight, flora and warm white fluorescent lamps at a ratio of 1:1:1, providing 130 µmol s^− 1^ m^− 2^ light intensity with a 16/8 h photoperiod.

### Data collection and statistical analysis

After the multiplication phase, the following parameters were recorded and calculated: shoot length (SL_M_, mm), number of new shoots per explant (SN_M_), node number per shoot (NN_M_), survival percentage (A_M_%), hyperhydration percentage (H_M_%), and flowering percentage (F_M_%). From the recorded data, a multiplication index (MI; Szarvas et al. [54]) and an optimal growth index for the multiplication phase (OGI_M_) were calculated as follows:

$$\:{OGI}_{M}=\left(\frac{{A}_{M}\%}{100}\right)*\left(1-\frac{{H}_{M}\%}{100}\right)*\left(1-\:\frac{{F}_{M}\%}{100}\right)$$

$$\:MI=\:\frac{{SL}_{M}*\:{SN}_{M}}{{NN}_{M}}$$

After the elongation phase, the shoot length (SL_E_, mm), node number per shoot (NN_E_), survival percentage (A_E_%), hyperhydration percentage (H_E_%), and flowering percentage (F_E_%) were recorded and calculated. From the above data, an elongation index (EI) and an optimal growth index for the elongation phase (OGI_E_) were calculated as follows:

$$\:{OGI}_{E}=\left(\frac{{A}_{E}\%}{100}\right)*\left(1-\frac{{H}_{E}\%}{100}\right)*\left(1-\:\frac{{F}_{E}\%}{100}\right)$$

$$\:EI=\:\frac{{SL}_{E}}{{NN}_{E}}$$

After the rooting phase, the root length (RL, mm), root number (RN), rooting percentage (RP%), survival percentage (A_R_%), hyperhydration percentage (H_R_%), and flowering percentage (F_R_%) were measured. From the above data, a rooting index (EI) and an optimal growth index for the rooting phase (OGI_R_) were calculated as follows:

$$\:{OGI}_{R}=\left(\frac{{A}_{R}\%}{100}\right)*\left(1-\frac{{H}_{R}\%}{100}\right)*\left(1-\:\frac{{F}_{R}\%}{100}\right)$$

$$\:RI=RN*RL*\left(\frac{RP\%}{100}\right)$$

The obtained morphological data from different phases of micropropagation were subjected to statistical analysis by one-way ANOVA followed by Tukey’s test (*p* < 0.05) using SPSS for Windows software (SPSS^®^, version 21.0). After acclimation, the number of plantlets that survived and developed at least one new leaf were counted, and a percentage was calculated.

## Data Availability

No datasets were generated or analysed during the current study.
